# Editorial: Advancing neurodegenerative disease biomarkers: the role of neuroimaging in TDP-43 and tau proteinopathies

**DOI:** 10.3389/fnins.2026.1879968

**Published:** 2026-06-18

**Authors:** Rodolfo G. Gatto, Hossam Youssef, Yuen Gao

**Affiliations:** 1 Department of Neurology, Mayo Clinic, Rochester, MN, United States; 2 Department of Radiology, Michigan State University, East Lansing, MI, United States

**Keywords:** molecular biomarkers, neurodegenerative diseases, neuroimaging, tau, TDP-43

**Figure 1:**
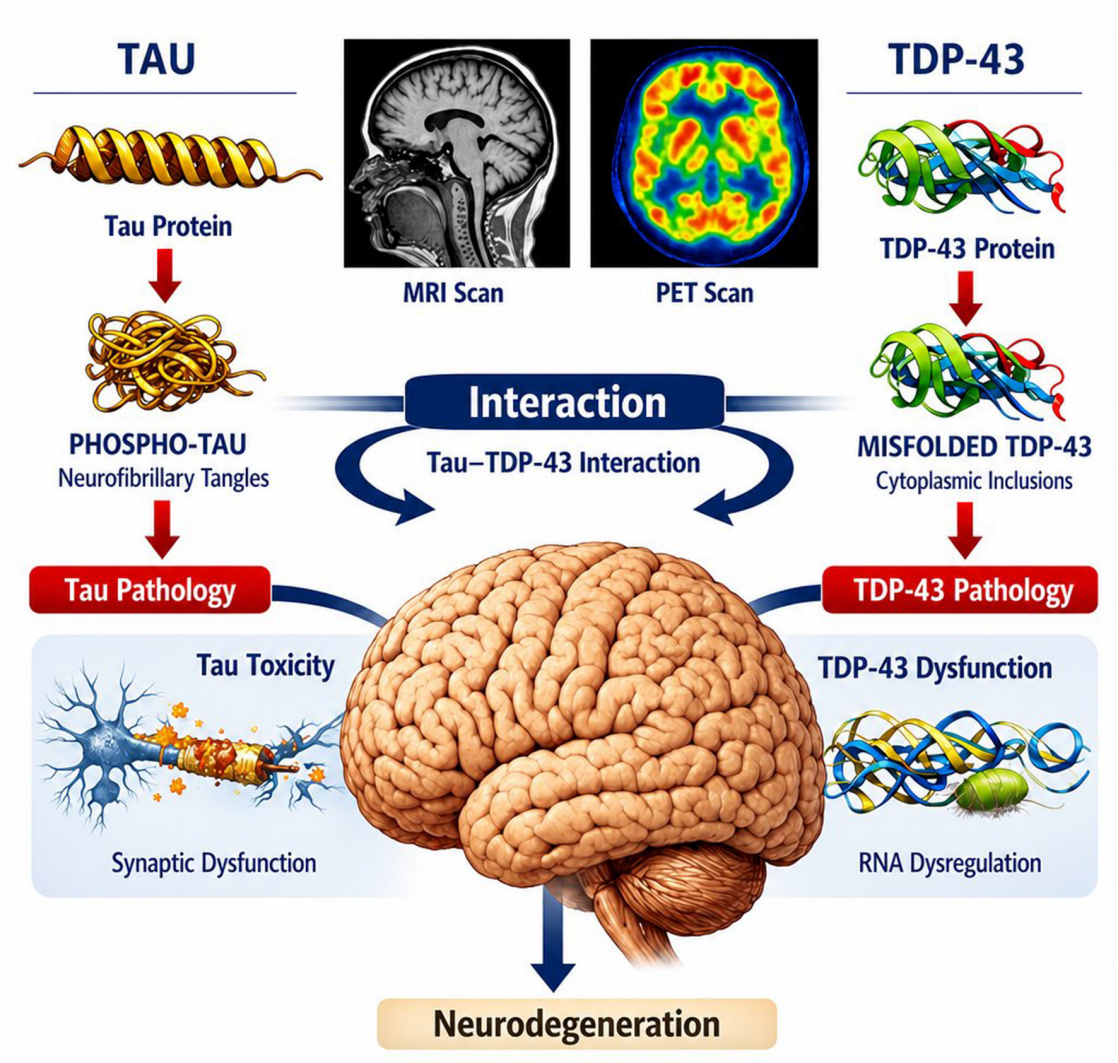
Conceptual model illustrating the potential simultaneous contributions of tau and TDP-43 (TAR DNA-binding protein 43) pathologies to neurodegeneration. Multimodal neuroimaging at the top center integrates structural magnetic resonance imaging (MRI), reflecting patterns of brain atrophy, and positron emission tomography (PET), capturing *in vivo* molecular pathology. On the **left**, tau undergoes abnormal post-translational modification and aggregates into neurofibrillary tangles, leading to tau pathology and synaptic dysfunction. On the **right**, TDP-43 misfolding and cytoplasmic accumulation give rise to TDP-43 pathology and ribonucleic acid (RNA) dysregulation. Bidirectional tau-TDP-43 interactions may amplify pathogenic mechanisms, ultimately converging and enhancing neurodegeneration.

The landscape of neurodegeneration research is undergoing a fundamental transformation, driven by a growing focus on TDP-43 and tau as central, interacting proteinopathies. These proteins recur across multiple neurodegenerative disorders and increasingly appear to act together, amplifying neuronal vulnerability and accelerating disease progression (Youssef et al., 2025). Rather than viewing them as independent entities, the field now recognizes that abnormal proteins often co-occur and may operate along a shared pathological axis (Figure 1). This perspective suggests that TDP-43 and tau influence each other's aggregation dynamics and enhance neurotoxicity, underscoring the importance of clarifying their molecular interplay (Nasir and Nouh).

In parallel, the scope of research is expanding to include additional phenotypic dimensions. Work by Avila-Villanueva et al. highlights sensory dysfunction as an early disease feature. Historically, impairments in smell, taste, and sensory processing were attributed to aging, but evidence now indicates these changes often precede cognitive symptoms. These early alterations may reflect tau deposition within sensory cortices. Recognizing sensory decline as part of the disease continuum enables earlier detection and broadens definitions of onset to include perception and behavior.

Neuroimaging advances are reshaping our understanding of disease progression. Diffusion tensor imaging (DTI) reveals that tau pathology can be inferred from microstructural disruption patterns. Detecting subtle changes early is redefining disease timelines and identifying new candidate biomarkers (Costa et al., 2025). Imaging staging systems now classify most patients with tauopathies such as progressive supranuclear palsy and track longitudinal change as shown by Bârlescu et al. More advanced diffusion approaches, including neurite orientation dispersion and density imaging, extend these capabilities further. NODDI provides refined measures of neurite density and organization and is applied across PSP cohorts (Gatto et al., 2024). In amyotrophic lateral sclerosis, exploratory work examined deep gray matter microstructure and identified nominal associations with clinical disability. Although these findings require validation, they underscore sensitivity to early alterations linked to tau and TDP-43.

Biomarker discovery is also evolving beyond imaging. Fluid-based measures are now explored as complementary tools capturing disease biology in clinical settings (Li et al., 2024). However, not all candidates ultimately prove informative. A longitudinal study by Aljuhani, examined tumor necrosis factor alpha and receptors across a decade of cerebrospinal fluid data and found no association with progression. These negative findings remain essential for refining hypotheses and emphasize the complexity of neuroinflammatory pathways. These results demonstrate the limitations of relying on single biomarkers and further support integrative strategies.

Accordingly, multimodal approaches can combine imaging and fluid measures to provide a broader perspective. Yu et al. integrated diffusion metrics, hippocampal diffusivity, and fluid profiles to characterize progression across healthy controls, mild cognitive impairment, and Alzheimer's disease (AD). Each modality adds a distinct perspective while contributing to a coherent disease trajectory. Diffusion along the perivascular space, reflecting glymphatic function, declines mainly at symptomatic stages. Hippocampal diffusivity captures earlier microstructural changes during mild impairment, whereas fluid markers reveal amyloid depletion and tau accumulation. Together, these combined measures illustrate how multimodal frameworks capture disease evolution across biological and temporal scales.

Computational methods add another important dimension. Machine learning models trained on imaging datasets identify patterns distinguishing tauopathy variants (Braun et al., 2025). However, several challenges remain. Clinical and cognitive measures signal short-term progression, but the rarity of events reduces predictive precision and increases false positives, as shown by Akinwumi et al. Although some models show promise, performance remains insufficient for routine use. These limitations clearly highlight the need for rigorous validation in independent cohorts. Nevertheless, machine learning offers a valuable framework for future precision medicine, especially as datasets become larger and more representative over time.

A unifying concept is network-based propagation of proteinopathies. Misfolded proteins such as tau and TDP-43 spread along interconnected neuronal networks. Subcortical regions, including the amygdala and deep gray matter, act as vulnerable hubs facilitating this propagation. The amygdala, with extensive connectivity, serves as a central convergence point for pathological processes. This network architecture may help explain early clinical manifestations (Sharbafshaaer et al.). Microstructural changes in the amygdala relate to fine motor decline, while sensory pathways connect directly to the amygdala and hippocampus, providing a substrate for early deficits (Avila-Villanueva et al.). Advanced neuroimaging now enables mapping of sequential regional changes *in vivo*, supporting propagation models across disease stages (Bârlescu et al.).

As datasets continue to expand and analytical tools improve, new opportunities will emerge to uncover relationships between pathological processes. Cross-disciplinary collaboration becomes increasingly essential, bringing together expertise in imaging, neuropathology, clinical neurology, and data science. Such collaboration supports development of robust biomarkers and reproducible models across diverse populations. Importantly, careful attention to negative findings and methodological limitations strengthens scientific rigor and prevents overinterpretation of preliminary observations. Balanced evaluation of the available evidence encourages realistic expectations for clinical translation and guides the design of future studies. Further studies in longitudinal cohorts, standardized protocols, and data sharing will accelerate discovery and validation of meaningful biomarkers.

Ultimately, the convergence of molecular, structural, and computational insights offers a realistic path toward precision medicine in neurodegenerative diseases. By identifying early pathological changes, clinicians may intervene before significant neuronal loss occurs. At the same time, improved prediction of individual trajectories can inform personalized care and optimize therapeutic strategies. Continued refinement of multimodal frameworks will further enhance sensitivity and specificity of disease detection.

As understanding deepens, translation into clinical practice will require careful validation, regulatory alignment, and seamless integration into healthcare systems. Education of clinicians and researchers will also be necessary to interpret increasingly complex biomarker profiles and implement data-driven decision tools responsibly. Ethical considerations, including data privacy and equitable access, must remain central throughout development and implementation.

Despite enduring challenges, the trajectory of the field remains clear. Integrated approaches are moving research beyond isolated observations toward a system-level understanding of disease. This shift creates opportunities to redefine diagnosis, monitor progression more accurately, and develop interventions targeting underlying biological mechanisms rather than symptoms alone. Together, these advances offer genuine promises for altering disease trajectories and improving patient outcomes in the coming years through sustained scientific collaboration and innovation across disciplines worldwide and beyond.
